# Mom2B: a study of perinatal health via smartphone application and machine learning methods

**DOI:** 10.1192/j.eurpsy.2022.1472

**Published:** 2022-09-01

**Authors:** A. Bilal, D. Bathula, E. Bränn, E. Fransson, J. Virk, F. Papadopoulos, A. Skalkidou

**Affiliations:** 1 Uppsala University, Neuroscience, Psychiatry, Uppsala, Sweden; 2 Indian Institute of Technology Ropar, Computer Science And Engineering, Punjab, India; 3 Uppsala University, Women’s And Children’s Health, Uppsala, Sweden

**Keywords:** peripartum depression, digital phenotyping data, deep learning models

## Abstract

**Introduction:**

Peripartum depression (PPD) impacts around 12% of women globally and is a leading cause of maternal mortality. However, there are currently no accurate methods in use to identify women at high risk for depressive symptoms on an individual level. An initial study was done to assess the value of deep learning models to predict perinatal depression from women at six weeks postpartum. Clinical, demographic, and psychometric questionnaire data was obtained from the “Biology, Affect, Stress, Imaging and Cognition during Pregnancy and the Puerperium” (BASIC) cohort, collected from 2009-2018 in Uppsala, Sweden. An ensemble of artificial neural networks and decision trees-based classifiers with majority voting gave the best and balanced results, with nearly 75% accuracy. Predictive variables identified in this study were used to inform the development of the ongoing Swedish Mom2B study.

**Objectives:**

The aim of the Mom2be study is to use digital phenotyping data collected via the Mom2B mobile app to evaluate predictive models of the risk of perinatal depression.

**Methods:**

In the Mom2B app, clinical, sociodemographic and psychometric information is collected through questionnaires, including the Edinburgh Postnatal Depression Scale (EPDS). Audio recordings are recurrently obtained upon prompts, and passive data from smartphone sensors and activity logs, reflecting social-media activity and mobility patterns. Subsequently, we will implement and evaluate advanced machine learning and deep learning models to predict the risk of PPD in the third pregnancy trimester, as well as during the early and late postpartum period, and identify variables with the strongest predictive value.

**Results:**

Analyses are ongoing.

**Conclusions:**

Pending results.

**Disclosure:**

No significant relationships.

